# Follow-Up Infarct Volume Prediction by CTP-Based Hypoperfusion Index, and the Discrepancy between Small Follow-Up Infarct Volume and Poor Functional Outcome—A Multicenter Study

**DOI:** 10.3390/diagnostics13010152

**Published:** 2023-01-02

**Authors:** Pengyu Zhou, Ran Li, Siyun Liu, Jincheng Wang, Lixiang Huang, Bin Song, Xiaoqiang Tang, Boyu Chen, Haiting Yang, Chengcheng Zhu, Ajay Malhotra, Yuting Wang

**Affiliations:** 1 Department of Radiology, Sichuan Provincial People’s Hospital, University of Electronic Science and Technology of China, Chengdu 610056, China; 2 GE Healthcare, Beijing 100176, China; 3 Department of Radiology, First Affiliated Hospital, College of Medicine, Zhejiang University, Hangzhou 310027, China; 4 Department of Radiology, Tianjin First Central Hospital, School of Medicine, Nankai University, Tianjin 300071, China; 5 Minhang Hospital, Fudan University, Shanghai 200437, China; 6 The Affiliated Changzhou No. 2 People’s Hospital of Nanjing Medical University, Changzhou 213003, China; 7 The First Affiliated Hospital, China Medical University, Shenyang 110122, China; 8 Lanzhou University Second Hospital, Lanzhou 730030, China; 9 Department of Radiology, University of Washington, Seattle, WA 98101, USA; 10 Department of Radiology and Biomedical Imaging, Yale School of Medicine, New Haven, CT 06501, USA

**Keywords:** follow-up infarct volume, large vessel occlusion, computed tomography, hypoperfusion index

## Abstract

(1) Background: Follow-up infarct volume (FIV) may have implications for prognostication in acute ischemic stroke patients. Factors predicting the discrepancy between FIV and 90-day outcomes are poorly understood. We aimed to develop a comprehensive predictive model of FIV and explore factors associated with the discrepancy. (2) Methods: Patients with acute anterior circulation large vessel occlusion were included. Baseline clinical and CT features were extracted and analyzed, including the CTP-based hypoperfusion index (HI) and the NCCT-based e-ASPECT, measured by automated software. FIV was assessed on follow-up NCCT at 3–7 days. Multiple linear regression was used to construct the predictive model. Subgroup analysis was performed to explore factors associated with poor outcomes (90-mRS scores 3–6) in small FIV (<70 mL). (3) Results: There were 170 patients included. Baseline e-ASPECT, infarct core volume, hypoperfusion volume, HI, baseline international normalized ratio, and successful recanalization were associated with FIV and included in constructing the predictive model. Baseline NIHSS, baseline hypertension, stroke history, and current tobacco use were associated with poor outcomes in small FIV. (4) Conclusions: A comprehensive predictive model (including HI) of FIV was constructed. We also emphasized the importance of hypertension and smoking status at baseline for the functional outcomes in patients with a small FIV.

## 1. Introduction

Clinical studies showed that the follow-up infarct volume (FIV) in the recanalization therapy group (thrombectomy or thrombolysis) was significantly smaller than in the control group in patients with acute ischemic stroke due to a large vessel occlusion (LVO) in the anterior circulation (including internal carotid artery, middle cerebral artery, and anterior cerebral artery), which seems to be explained by the salvage and preservation of brain tissue [1,2,3]. Although many randomized trials employed the functional outcome at 90 days as the primary outcome, a recent meta-analysis showed a significant association between the extent of FIV and the 90-day functional outcome [4]. Unlike assessing outcomes at 90 days, FIV is easy to access and has the potential to serve as a surrogate marker for outcome measurement in clinics and trials and therefore becomes of great clinical concern.

Factors associated with FIV have been explored, such as demographic characteristics, baseline Alberta Stroke Program Early Computed Tomography Score (ASPECT), infarct core volume, and hypoperfusion volume measured on computed tomography perfusion (CTP). However, manual scoring of ASPECT often suffers from poor interrater reliability [5,6]. ASPECT scored by automated software (e-ASPECT) with potentially higher reproducibility, however, has not been previously assessed for FIV estimation. An automated software NovoStroke Kit (NSK, research-only prototype, not cleared for clinical use, GE Healthcare, China), which uses deep learning-based brain tissue segmentation for advanced imaging analysis, has been shown to solve the poor interrater reliability in manual ASPECT scoring [7], showing high consistency with senior neuroradiologists and RAPID software in the assessment of ASPECT [8].

A recent study showed that the semi-quantitative CTA whole-brain collateral circulation vascular score is associated with FIV [9]. However, it requires additional sophisticated evaluations and could be observer dependent. The role of collateral status as automatically evaluated by the quantitative parameter hypoperfusion index (HI) on CTP in predicting FIV has not been well assessed.

Previous studies have shown that a small infarct volume (less than 70 mL) is generally associated with a good prognosis [10,11]. Nevertheless, patients who have poor prognoses despite small infarct volume are not uncommon clinically. It is greatly needed to evaluate the incidence of such discrepancy and the associated factors.

In summary, we aim to (1) develop a comprehensive predictive model of FIV using clinical and imaging features, including e-ASPECT and HI; (2) explore the factors associated with poor functional outcomes in small FIV patients.

## 2. Materials and Methods

### 2.1. Study Design and Patients

This was a multicenter study. We retrospectively collected 211 patients with suspected acute anterior circulation LVO admitted to 7 stroke centers from February to June 2021. The institutional review board approved this study, with a waiver of informed consent for this retrospective study. All procedures were performed in accordance with local and federal regulations and the Declaration of Helsinki. The inclusion criteria were as follows: (1) The time from the last known-well to admission was less than 24 h. (2) Baseline one-stop CT, including non-contrast CT (NCCT) and CTP, with or without CT angiography (CTA). Digital subtraction angiography (DSA) was an alternative when CTA was not possible. (3) Acute anterior circulation LVO. (4) Follow-up NCCT at 3–7 days post-presentation and the functional outcome measurement on modified Rankin Scales at 90 days (90d-mRS) after therapy or admission. The exclusion criteria were as follows: (1) Incomplete baseline one-stop CT data or with poor quality. (2) Patients with a definite parenchymal hemorrhage. (3) Missing follow-up NCCT at 3–7 days or 90d-mRS.

### 2.2. Demographics and Clinical Risk Factors

Patient demographics and clinical risk factors at baseline were obtained from the electronic clinic records. Hypertension was defined as systolic blood pressure values ≥ 140 mmHg and/or diastolic blood pressure values ≥ 90 mmHg [12]. Diabetes was defined as a self-reported history of diabetes or diagnosed by the guidelines of the American Diabetes Association [13]. History of stroke, including ischemic stroke or intracerebral hemorrhage stroke, was recorded. Current tobacco use (smoked ≥ 1 cigarette daily during the past year) and current alcohol use (consumed any dose of alcohol daily during the past year) were also recorded. Presenting NIHSS score was determined at baseline, with higher scores reflecting increased clinical stroke severity. Successful recanalization was defined as an mTICI (modified Thrombolysis in Cerebral Infarction) score of 2b to 3 on intraoperative DSA or an mAOL (modified Arterial Occlusive Lesion) score of 3 on CTA at 24 h [14]. 90d-mRS score was obtained by a senior neurologist, who was blind to the patient clinic and imaging information, using follow-up telephone calls. The good and poor functional outcomes were defined as scores of 0–2 and 3–6 on 90d-mRS, respectively.

### 2.3. Image Processing and Analysis

CT data was acquired by one-stop CT protocol, including NCCT and CTP, with or without CTA. It was required that all participating centers employed the following basic scan parameters to ensure the acquired studies were comparable: 100–120 kV; 120–420 mA; FOV,320; section thickness of 1.25 mm for NCCT and 5.00 mm for CTP; reconstruction interval, 0.5 mm; scanning direction from the second cervical vertebra to the vertex. All NCCT and CTP images were automatically analyzed using the NSK software. The e-ASPECT (ASPECT by NSK) and the manual-ASPECT were obtained from baseline NCCT. For perfusion analysis, baseline CTP images were processed by using the conventional deconvolution method. Perfusion-related parametric maps were obtained, including cerebral blood flow (CBF), cerebral blood volume (CBV), and time-to-maximum (Tmax) of the residue function. Finally, volume-related parameters were calculated based on setting certain thresholds on the perfusion maps, such as infarct core volume (the lesion volume of rCBF < 30%), hypoperfusion volume (the lesion volume of Tmax > 6 s), ischemic penumbra volume (the difference between the hypoperfusion volume and the infarct core volume), and HI (the lesion volume of Tmax > 10 s divided by the lesion volume of Tmax > 6 s). FIV was measured on follow-up NCCT at 3–7 days after therapy or admission manually, using the (A × B × C)/2 formula [15]. Measurements of the manual-ASPECT and FIV were independently completed by two trained observers. The mean values of the manual-ASPECT and FIV were calculated respectively and used for later analysis. To assess the reliability and clinical applicability of the e-ASPECT, the relatively stable mean scoring of manual-ASPECT was used as the reference.

### 2.4. Statistics

The Shapiro–Wilk test was used to ensure the normality of continuous variables. Categorical variables, such as clinical and treatment characteristics of the study population, were presented as counts with proportion. Continuous variables were presented as median with interquartile range (IQR) due to the non-normal distribution of the data. Multivariate linear regression analysis was performed to determine the independent predictors of FIV, and a prediction model was constructed. We distinguished the large and the small FIV (<70 mL) and divided them into two subgroups using the 70 mL threshold [11]. Receiver operator characteristic (ROC) analysis was applicated to evaluate the performance of the prediction model. The corresponding area under the ROC curve (AUC) and the 95% confidence interval were reported as well. Youden index was performed to determine the best cutoff value of the prediction model, and the related sensitivity and specificity were recorded. Discrepancies were assessed between good and poor functional outcomes with a small FIV using nonparametric Mann–Whitney U tests for continuous variables and Chi-square test or Fisher’s Exact tests for categorical variables. Statistical correlation significance for FIV and 90d-mRS was assessed by Spearman’s rank correlation coefficient (rs). Intraclass correlation coefficient (ICC) was used to assess the consistency between e-ASPECT and manual-ASPECT mean scoring. Wilcoxon analysis was performed to assess the variability of non-normally distributed data. Consistency and variance analysis of the results from the two observers was also carried out. The default calculated positive and negative values for dichotomous classification in the predictive model were 1 and 0, respectively. Statistical analyses were performed using SPSS version 23.0 (IBM Corp., Chicago, IL, USA), and graphic results were drawn using GraphPadPrism 5 (GraphPad Software Inc., San Diego, CA, USA). A *p*-value < 0.05 was considered statistically significant.

## 3. Results

**Figure 1:**
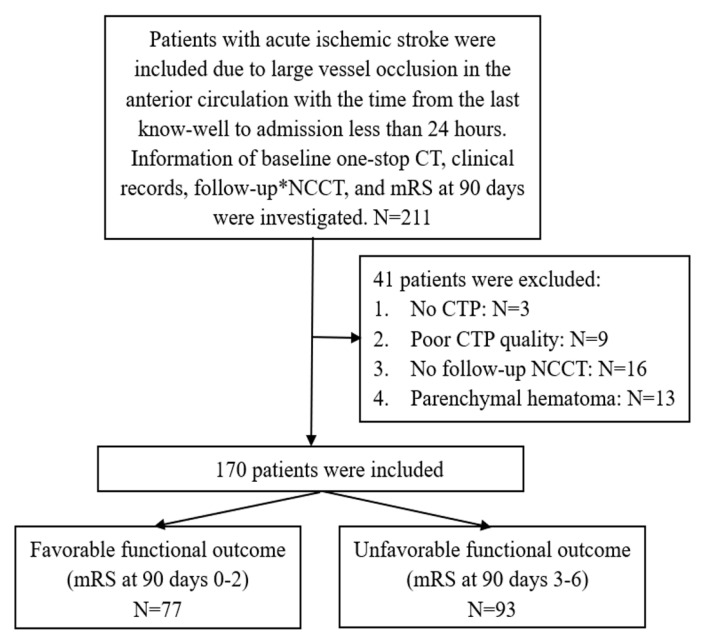
Flowchart describing the inclusion and exclusion criteria of this study; one-stop CT (including NCCT, CTP, and CTA); * 3–7 days after endovascular treatment or admission.

Forty-one patients were excluded: 12 patients had incomplete or suboptimal baseline one-stop CT, 13 patients had a definite parenchymal hemorrhage, and 16 patients had no follow-up NCCT within 3–7 days. The flowchart of this study is presented in Figure 1.

Among the 170 patients included, the median (IQR) age was 68 (57–76) years, 60 patients (35.3%) were women, the median (IQR) baseline NIHSS was 11 (6–18), and the median (IQR) time from the last known-well to the hospital was 5 (2–9.3) hours. Thrombectomy and thrombolysis were performed in 114 (67.1%) and 23 (13.5%) patients, respectively. Four patients (2.3%) received balloon stent dilatation. Twenty-nine patients (17.1%) received conservative treatment (supportive medication). Successful recanalization was obtained in 111 patients (65.3%). Good functional outcomes (90d-mRS 0–2) were obtained in 77 patients (45.3%). Patient characteristics are shown in Table 1. There was an excellent consistency (ICC = 0.821, *p* < 0.01) between e-ASPECT and manual-ASPECT at baseline, as shown in Table 2.

### 3.1. Predictive Model Construction

The median (IQR) FIV was 54.2 (11.9–134.7) mL. Baseline e-ASPECT, infarct core volume, hypoperfusion volume, HI, international normalized ratio (INR) at admission, and successful recanalization were the independent predictors of FIV.

The model was as follows:

FIV = Baseline e-ASPECT × (−11.74) + infarct core volume × 0.34 + hypoperfusion volume × 0.17 + HI × 89.26 + INR × (−88.62) + successful recanalization × (−51.45) + 224.35(1)

**Figure 2:**
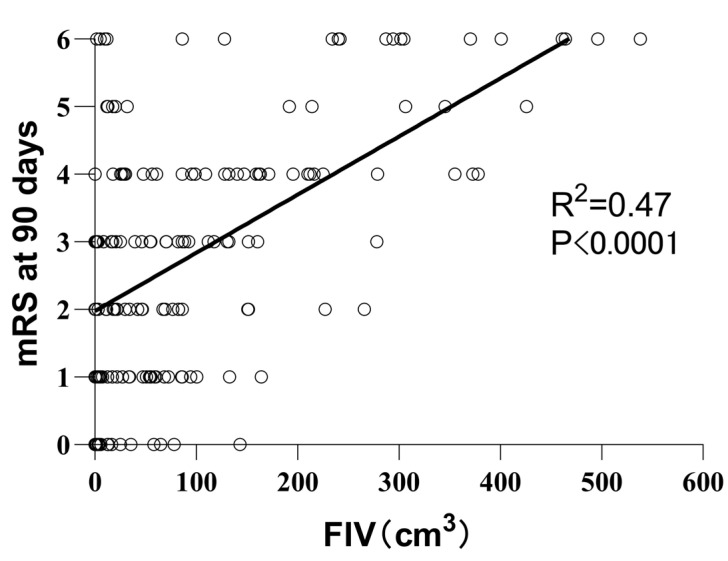
The line chart of Spearman’s rank correlation results between FIV and mRS at 90 days; FIV = follow-up infarct volume at 3–7 days. Each circle represents a patient.

The detailed results are shown in Table 3. The AUC of this model to distinguish a large FIV (≥70 mL) was 0.851 (95% CI, 0.794–0.909), with a 76.4% sensitivity and 82.7% specificity. There was a moderate statistical correlation between FIV and 90d-mRS (rs = 0.474, *p* < 0.01), which is shown in Figure 2.

### 3.2. The Discrepancy Associated with Poor Functional Outcomes in Small FIV

**Figure 3:**
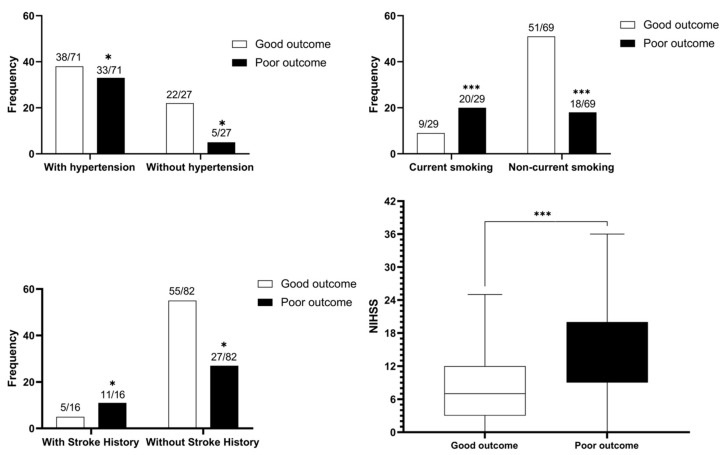
The column charts and boxplot results of significant factors related to a poor outcome in small (<70ml) FIV; Good outcome (mRS 0-2) and poor outcome (mRS 3-6). *: *p* < 0.05; ***: *p* < 0.001.

In the small FIV group of 98 patients, 38 patients (38.8%) had poor functional outcomes and 60 patients (61.2%) had good functional outcomes. Baseline NIHSS, baseline hypertension, history of stroke, and current tobacco use were significantly different in the two subgroups. The detailed results are shown in Table 4. The column charts and boxplot results of significant factors related to a poor outcome in small FIV are shown in Figure 3. Compared with the good functional outcome subgroup, higher baseline NIHSS (median, 13.5 versus 7, *p* < 0.001), baseline hypertension (OR, 3.82; 95% CI, 1.30–11.22), history of stroke (OR, 4.81; 95% CI, 1.42–14.20), and current tobacco use (OR, 6.30; 95% CI, 2.43–16.32) were likely related to poor functional outcomes despite small FIV, regardless of the therapeutic regimens.

## 4. Discussion

In this multicenter, retrospective study involving comprehensive stroke-related parameters and advanced automated stroke analysis software, we found more independent predictive factors (including HI) for FIV than in previous studies [16,17,18,19,20,21], and we present the performance results of the subsequent prediction model. This model showed a good prediction ability for large FIV. In addition, we explored the factors significantly associated with poor prognoses in patients with a small FIV.

### 4.1. Predictive Models for FIV

FIV was determined as an objective prognostic measurement of clinical outcomes [22,23]. In reality, infarct evolution is a dynamic process. Studies showed that growth of infarct volume was common in the first 24 h post-stroke onset, even with a successful recanalization [24,25]. Therefore, an accurate prediction merely by baseline CTP analysis has inherent constraints.

HI, as automatically calculated at baseline from CTP, correlates well with the quality of collateral circulation, and was associated with FIV growth and functional outcome in previous studies [26,27,28]. Our results demonstrated HI to be an independent predictor of FIV and emphasized the importance of quantitative collateral circulation-related parameters in the prediction of FIV.

We also found INR to be negatively correlated with the FIV, which was consistent with the findings of Matsumoto et al. [29]. They found the infarct volume of the INR ≥ 2.00 group was significantly smaller than in the other INR groups (INR < 1.60 and 1.60–1.99) and a trend toward smaller infarct volumes with larger INR.

In recent years, artificial intelligence technology such as deep learning has been used to construct a predictive model for infarct core volume [30,31] or FIV [21]. These studies have incorporated native CTP images and treatment parameters into their models, such as native CTP, CBV, CBF, MTT (Mean transit time), Tmax, infarction location, and the times related to stroke, showing good prediction performance. However, these models did not fully incorporate clinical features and the collateral status measured by CTP in terms of HI. Moreover, Zhu et al. and Kasasbeh et al. both aimed to predict infarct core volume at baseline, not the FIV [30,31].

Previous studies considered FIV 24-h post-stroke onset as an important prognostic marker, which was used as a radiological endpoint in research [32,33]. Because of the common FIV growth at 24-h post-stroke onset, assessing the FIV at this subacute time frame was challenging, as this coincides with the period of maximal cerebral edema, which could cause substantial overestimation of infarct volume [24,25]. Since cerebral edema starts to resolve in the latter subacute period, Christensen et al. suggested that target mismatch (at-risk penumbral tissue) may be present up to 48-h post-stroke onset and potentially contribute to delayed infarct expansion in the subacute period [34]. Therefore, we chose to measure the infarct volume at the time point of 3–7 days post-stroke onset to avoid the impact of edema to the extent possible.

### 4.2. The Discrepancy Associated with Poor Functional Outcomes in Small FIV

Issues regarding FIV are of potential clinical concern, because it has been suggested as a surrogate marker for outcome measurement in clinics and trials. Unlike assessing outcomes at 90 days, which relates to certain rates of loss to follow-up, FIV can be readily assessed in routine imaging before discharge. However, patients who had poor outcomes at 90 days despite small FIV are not uncommon in clinical practice, and it is greatly needed to evaluate the incidence of such a condition and the factors associated with it. Aravind et al. segmented FIV by quartiles and found that discrepancies between functional outcomes and small post-EVT infarct volume (i.e., ≤25th percentile) were age, vascular risk factors, comorbidities, and post-treatment complications [35]. Our findings are partially consistent with theirs. There were 38 (38.8%) patients who had poor outcomes in 98 patients with a small FIV, and a moderate statistical correlation was found between FIV and 90d-mRS in our study. We found that the discrepancies between small FIV and functional outcomes are related to vascular risk factors, pretreatment stroke severity (baseline NIHSS), and comorbidity (hypertension). This may explain the relatively low predictive value for the 90-day outcomes, which was incompletely captured by FIV. It also highlights the importance of blood pressure and smoking status at baseline in accounting for the outcomes.

### 4.3. NSK Software

**Figure 4:**
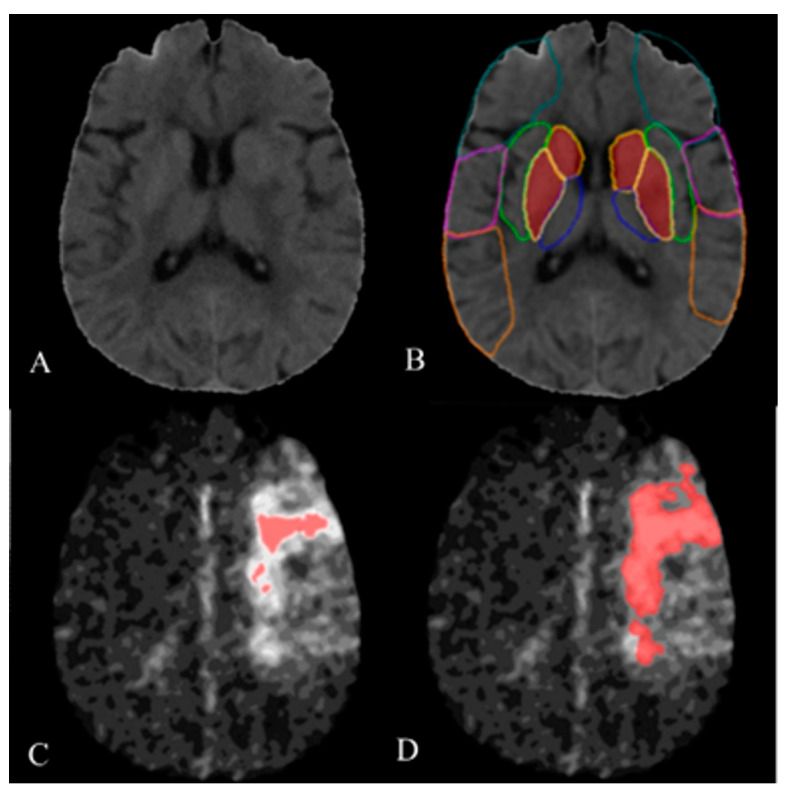
The schematic diagrams of the calculation of e-ASPECT and HI using NSK software; ABCD are obtained from the same patient with a left middle cerebral artery occlusion. (**A**) NCCT; (**B**) ASPECT is measured using NSK software (e-ASPECT), and the calculated score is 8; (**C**) the volume of Tmax > 10 s (marked red), and the calculated volume is 6.46 mL; (**D**) the volume of Tmax > 6 s (marked red), and the calculated volume is 48.40 mL. The HI of this patient is 0.13.

The schematic diagrams of the calculation of e-ASPECT and HI using NSK software are shown in Figure 4. Several commercially available software applications can perform automated stroke-related parameter evaluation, such as RAPID, Syngo software (Syngo. via CT Neuro Perfusion VB30; Siemens Healthineers, Erlangen, Germany), etc. [36]. Most of these applications adopt machine learning or deep learning methods. NSK employs deep learning methods and has shown good agreement with the expert consensus for NCCT within 6 h after stroke onset; it increased to an excellent agreement at the time window after 6 h [8]. The software is available for use in all of this study’s participating centers. Our results also showed the e-ASPECTs had excellent consistency with the manual-ASPECTs.

### 4.4. Future Directions

The role of HI in predicting different atlas-based patterns of FIV requires further investigation. Recent studies have shown that infarct patterns and volumes correlate with prognosis [37,38]. Marielle et al. developed an automatic atlas-based segmentation for the brain under CT scans, showing that the increase of FIV in brain areas of high mRS relevance was independently associated with unfavorable functional outcomes [39]. Future analyses could include more detailed and precise classification to examine how eloquence in affected regions (e.g., the internal capsule and white matter in small infarcts) or spared regions (e.g., the unaffected motor cortex in large infarcts) predicts FIV and explains the differences between FIV and mRS.

### 4.5. Limitations

First, the sample size was relatively small, which may be partly explained by the rigorous inclusion criteria and required parameters of image acquisition for obtaining comparable image data. Second, we used NCCT as the follow-up tool rather than MRI, although MRI is generally considered to be the gold standard for infarct volume measurement and infarct pattern determination. However, the application of MRI is limited in many primary hospitals as well as in emergency settings, while CT is more widely available. Third, intracranial vascular variants were not considered, such as the azygous anterior cerebral artery, which is a normal variant in the circle of Willis.

## 5. Conclusions

Baseline e-ASPECT, infarct core volume, hypoperfusion volume, HI, INR at admission, and successful recanalization were the independent predictive factors of FIV. We also emphasize the importance of hypertension and smoking status at baseline for the functional outcomes in patients with FIV < 70 mL. The effects of blood pressure control and smoking cessation in the recovery period on functional prognosis are worth further investigation.

## Figures and Tables

**Table 1:** Demographic characteristics, treatments, and outcomes of the included patients.

	Median (IQR) or Percentage (N)
Age, years	68 (57–76)
Female	35.3% (60/170)
Hypertension	71.2% (121/170)
Diabetes	29.4% (50/170)
History of stroke	15.9% (27/170)
Current tobacco use	34.7% (59/170)
Current alcohol use	18.2% (31/170)
Baseline NIHSS	11(6–18)
The time from the last known-well to the hospital, hours	5.0 (2.0–9.3)
Occlusion locations	
ICA	30.0% (51/170)
MCA	67.1% (114/170)
ACA	2.9% (5/170)
Affected hemisphere	
Right	47.1% (80/170)
Left	50.6% (86/170)
Bilateral	2.3% (4/170)
Baseline e-ASPECT ^●^	7 (5–9)
Baseline manual-ASPECT	7 (5–9)
Infarct core volume ^○^, mL	11.0 (3.8–43.8)
Hypoperfusion volume ^◆^, mL	147.5 (76.5–243.3)
Ischemic penumbra volume ^◇^, mL	92.5 (44.5–193.8)
FIV, mL	54.2 (11.9–134.7)
Hypoperfusion index ^★^	0.25 (0.10–0.50)
Tmax8/6 ^☆^	0.52 (0.32–0.70)
Therapeutic regimens	
Thrombectomy	67.1% (114/170)
Thrombolysis	13.5% (23/170)
Conservative treatment	17.1% (29/170)
Balloon stent dilatation	2.3% (4/170)
Successful recanalization ^#^	65.3% (111/170)
Successful recanalization in EVT	78.1% (107/137)
Blood platelet	200.8 (150.8–235.5)
International normalized ratio	1.01 (0.96–1.07)
90 days mRS 0–2	45.3% (77/170)

IQR = interquartile range; FIV = follow-up infarct volume; EVT = endovascular treatment including thrombectomy and thrombolysis; ^○^ Volume of rCBF < 30%; ^●^ ASPECT score measured using an automatic software; ^◆^ Volume of Tmax > 6 s; ^◇^ The difference between the hypoperfusion volume and the infarct core volume; ^★^ The lesion volume of Tmax > 10 s divided by the lesion volume of Tmax > 6 s; ^☆^ The lesion volume of Tmax > 8 s divided by the lesion volume of Tmax > 6 s; ^#^ mTICI score was 2b to 3 on intraoperative DSA or the modified Arterial Occlusive Lesion score was 3 on CTA at 24 h.

**Table 2:** The consistent results in e-ASPECT, manual-ASPECT, and FIV.

Variable	Intraclass Correlation Coefficient	Spearman’s Rank Correlation Coefficient, *p*-Value	Wilcoxon Analysis of Statistical Values, *p*-Value
Manual-ASPECT in two observers	0.535	0.563, *p* < 0.01	Z = 4.676, *p* < 0.001
e-ASPECT ^●^ and manual-ASPECT mean scoring	0.821	0.744, *p* < 0.01	Z = 0.521, *p* = 0.605
FIV in two observers	0.921	0.870, *p* < 0.01	Z = 0.356, *p* = 0.722

FIV = follow-up infarct volume; ^●^ ASPECT score measured using automatic software.

**Table 3:** Uni- and multi-variate analysis using the linear mixed effect model for FIV.

FIV	Univariate Analysis			Multivariate Analysis		
	Estimate	SD	*p*-Value	Estimate	SD	*p*-Value
Constant	-	-	-	224.347	51.577	0.000 **
Gender	20.819	16.188	0.200	-	-	-
Age	0.578	0.535	0.281	-	-	-
Hypertension	5.862	15.673	0.709	-	-	-
Diabetes	30.520	18.829	0.055 ^	21.364	14.982	0.156
Current tobacco use	−24.046	16.916	0.157			
Current alcohol use	−17.137	19.248	0.375			
History of stroke	−25.141	19.309	0.195			
The time from the last known-well to the hospital	−1.486	1.158	0.201	-	-	-
Baseline NIHSS	1.165	0.918	0.207	-	-	-
Affected hemisphere	−3.809	13.143	0.772			
Occlusion locations	3.664	13.940	0.793	-	-	-
Therapeutic regimens	−15.917	9.710	0.103	-	-	-
Successful recanalization ^#^	−68.326	17.909	0.000 ^	−51.449	14.508	0.001 **
Baseline e-ASPECT ^●^	−9.879	3.128	0.002 ^	−11.736	2.882	0.000 **
Infarct core volume ^○^, mL	0.365	0.173	0.037 ^	0.337	0.170	0.048 *
Hypoperfusion volume ^◆^, mL	0.166	0.061	0.007	0.174	0.058	0.003 *
Ischemic penumbra volume ^◇^, mL	-	-	-	-	-	-
Blood platelet	0.082	0.111	0.460	-	-	-
International normalized ratio	−79.472	43.821	0.072 ^	−88.615	41.927	0.036 *
Hypoperfusion index ^★^	172.953	66.682	0.010 ^	89.262	33.808	0.009 *
Tmax8/6 ^☆^	−83.681	64.991	0.200	-	-	-

^ *p* < 0.1, * *p* < 0.05, ** *p* < 0.001; SD = standard deviation; FIV = follow-up infarct volume; ^#^ mTICI score was 2b to 3 on intraoperative DSA or the modified Arterial Occlusive Lesion score was 3 on CTA at 24 h; ^●^ ASPECT score measured using an automatic software; ^○^ Volume of rCBF < 30%; ^◆^ Volume of Tmax > 6 s; ^◇^ The difference between the hypoperfusion volume and the infarct core volume, which was excluded because of its collinearity with the hypoperfusion volume; ^★^ The lesion volume of Tmax > 10 s divided by the lesion volume of Tmax > 6 s; ^☆^ The lesion volume of Tmax > 8 s divided by the lesion volume of Tmax > 6 s.

**Table 4:** The discrepancies between different functional outcomes in FIV < 70 mL.

	90 Days mRS of 0–2 in FIV < 70 mL	90 Days mRS of 3–6 in FIV < 70 mL	*p*-Value, OR (95% CI)
Variable	Median (IQR) or Percentage (N)	Median (IQR) or Percentage (N)	
N	60	38	-
Age, years	66 (56–74)	67 (57–76)	0.64
Female	61.7% (37/60)	36.8% (14/38)	0.53
Hypertension	63.3% (38/60)	86.8% (33/38)	0.01 *3.82 (1.30–11.22)
Diabetes	21.7% (13/60)	28.9% (11/38)	0.28
History of stroke	8.3% (5/60)	28.9% (11/38)	0.01 *4.48 (1.42–14.20)
Current tobacco use	15.0% (9/60)	52.6% (20/38)	0.00 *6.30 (2.43–16.32)
Current alcohol use	11.7% (7/60)	18.4% (7/38)	0.26
Baseline NIHSS	7 (3–11)	13.5 (7.5–18.0)	0.00 **
The time from the last known-well to the hospital, hours	5.0 (2.6–12.4)	4.5 (2.0–8.0)	0.35
Occlusion locations	-	-	0.41
ICA	23.3% (14/60)	31.6% (12/38)	-
MCA	71.7% (43/60)	65.8% (25/38)	-
ACA	5% (3/60)	2.6% (1/38)	-
Affected hemisphere	-	-	0.46
Right	46.7% (28/60)	47.4% (18/38)	-
Left	51.7% (31/60)	52.6% (20/38)	-
Bilateral	1.7% (1/60)	0	-
Baseline e-ASPECT ^●^	8.5 (7.00–10.00)	7.50 (5.75–9.00)	0.09
Infarct core volume ^○^, mL	5.0 (1.0–12.8)	6.00 (1.00–17.25)	0.82
Hypoperfusion volume ^◆^, mL	88.0 (47.0–162.3)	137.5 (49.5–269.8)	0.08
Ischemic penumbra volume ^◇^, mL	73.0 (40.5–142.8)	133.5 (31.0–257.0)	0.10
Hypoperfusion index ^★^	0.19 (0.06–0.33)	0.20 (0.06–0.40)	0.55
Tmax8/6 ^☆^	0.44 (0.25–0.59)	0.44 (0.22–0.66)	0.62
FIV, mL	14.69 (3.02–45.18)	16.76 (2.60–26.10)	0.80
Therapeutic regimens	-	-	0.21
Thrombectomy	70.0% (42/60)	65.8% (25/38)	-
Thrombolysis	20% (12/60)	10.5% (4/38)	-
Conservative treatment	6.7% (4/60)	21.1% (8/38)	-
Balloon stent dilatation	3.3% (2/60)	2.6% (1/38)	-
Successful recanalization ^#^	83.3% (50/60)	71.1% (27/38)	0.37
Blood platelet	200.8 (151.25–242.25)	200.94 (139.50–233.25)	0.67
International normalized ratio	1.02 (0.96–1.08)	1.00 (0.95–1.06)	0.18

* *p* < 0.05, ** *p* < 0.001; IQR = interquartile range; FIV = follow-up infarct volume; ^●^ ASPECT score measured using an automatic software; ^○^ Volume of rCBF < 30%; ^◆^ Volume of Tmax > 6 s; ^◇^ The difference between the hypoperfusion volume and the infarct core volume; ^★^ The lesion volume of Tmax > 10 s divided by the lesion volume of Tmax > 6 s; ^☆^ The lesion volume of Tmax > 8 s divided by the lesion volume of Tmax > 6 s; ^#^ mTICI score was 2b to 3 on intraoperative DSA or the modified Arterial Occlusive Lesion score was 3 on CTA at 24 h.

## Data Availability

For any detailed research data, please contact the corresponding author.
